# Preperitoneal herniation as a complication of tansabdominal preperitoneal patch plasty: a report of two cases

**DOI:** 10.1186/s12893-021-01225-z

**Published:** 2021-05-01

**Authors:** Zhenyu Zou, Yilin Zhu, Fan Wang, Jinxin Cao, Yuchen Liu, Huiqi Yang, Minggang Wang

**Affiliations:** Department of Hernia and Abdominal Wall Surgery, Beijing Chaoyang Hospital, Capital Medical University, No. 5 Jingyuan Road, Shijingshan District, Beijing, 100043 China

**Keywords:** Inguinal hernia, Small bowel obstruction, Laparoscopy, Postoperative complication

## Abstract

**Background:**

Preperitoneal herniation is a rare complication after transabdominal preperitoneal patch plasty (TAPP) and may be caused by inadequate peritoneal closure. We herein report two cases of postoperative small bowel obstruction due to preperitoneal herniation through a disrupted peritoneum.

**Case presentation:**

Two men in their 70s were admitted to our center because of small bowel obstruction after TAPP. After examinations and unsuccessful conservative treatment, emergency laparoscopic exploration was performed. Preperitoneal herniation through the disrupted peritoneum was found. The herniated small bowel was reduced and the peritoneum was properly reclosed. The patients recovered and were discharged with normal bowel function.

**Conclusions:**

Inadequate peritoneal closure may cause preperitoneal herniation and lead to postoperative small bowel obstruction and even death. Hernia surgeons can avoid this complication by improving their suture technique and paying attention to the procedure details.

## Background

Inguinal hernia repair is one of the most common general surgery operations worldwide [1]. Compared with open repair, the advantages of laparoscopic inguinal hernia repair (LIHR) include decreased postoperative pain, faster recovery, a shorter hospital stay, better cosmesis, easier repair of recurrent hernias, and the ability to treat bilateral hernias concurrently [2–5]. However, reports of postoperative complications after LIHR have gradually increased [5–12].

Postoperative complications after LIHR include chronic pain; seroma formation; neurovascular injury; visceral injury; mesh infection, migration, and erosion; hernia recurrence; and testicular complications. Small bowel obstruction (SBO) is an uncommon complication of transabdominal preperitoneal patch plasty (TAPP), occurring with an estimated incidence of 0.2–0.5% [10]. The most common causes of SBO after TAPP include preperitoneal herniation (PH) through inadequate closure of the peritoneum and port sites as well as adhesion formation [10]. Every hernia surgeon must be aware of these potential causes of SBO, and technical failure should be avoided.

In this report, we present two cases of postoperative SBO caused by PH through a disrupted peritoneum after TAPP. This is a rare but fatal complication after a commonly performed procedure. This report presents our experience with the diagnosis, treatment, and prevention of this complication.

## Case presentation

### Case 1

A 76-year-old man presented with a recurrent right inguinal hernia after an open repair with a plug and patch had been performed at a secondary hospital 4 years previously.

Shortly after his admission to our hospital, TAPP was performed. During the procedure, we found that the plug was protruding from Hesselbach triangle into the abdominal cavity. A defect was identified lateral to the inferior epigastric vessels, and a right recurrent L2 hernia (European Hernia Society groin hernia classification system [13]) was diagnosed. The fusiform incision of the peritoneum was continued superior to the edge of the plug and down to the anterior superior iliac spine. Part of the plug was removed to create a bed for the mesh. The indirect hernia sac was completely isolated from the cord structures. A 10- × 15-cm piece of lightweight polypropylene mesh (TransEasy Medical Tech. Co., Ltd., Beijing, China) was implanted and fixed to the iliopubic tract, rectus muscle, and surface of the remaining plug using ProTack (Medtronic, Minneapolis, MN, USA). The peritoneal flap was closed with a running suture.

During the following 2 weeks, the patient experienced intermittent right lower abdominal pain and distension without nausea or vomiting. After conservative treatment including nasogastric decompression, lavage, and intravenous fluids, his symptoms were slightly relieved. On postoperative day 15, however, his right lower abdominal pain gradually worsened. Computed tomography (CT) scans of the abdomen and pelvis revealed air-fluid levels, small bowel distension, and exudates in the right groin area.

The patient underwent emergency laparoscopy. Pneumoperitoneum was created by insertion of a Veress needle into the left upper quadrant, 2 to 3 cm below the costal margin and away from the prior trocar sites. Additional ports were placed under direct vision. Inspection of the peritoneal cavity showed that the right peritoneum was disrupted and approximately 20 cm of small bowel had herniated through the defect. The small bowel loops were incarcerated in the preperitoneal space and adhered to the mesh. Because we suspected adhesions between the bowel and the tacks, conversion to open surgery was performed. An 8-cm incision was made in the right inguinal region. After removing part of the mesh, the abdominal cavity was entered. The bowel was uneventfully detached from the mesh and reduced into the abdominal cavity. The peritoneal flap was closed with an absorbable running suture, and a subcutaneous drain was placed. After tolerating a regular diet and demonstrating normal bowel function, the patient was discharged home the following week. He developed no further complications.

### Case 2

**Fig. 1:**
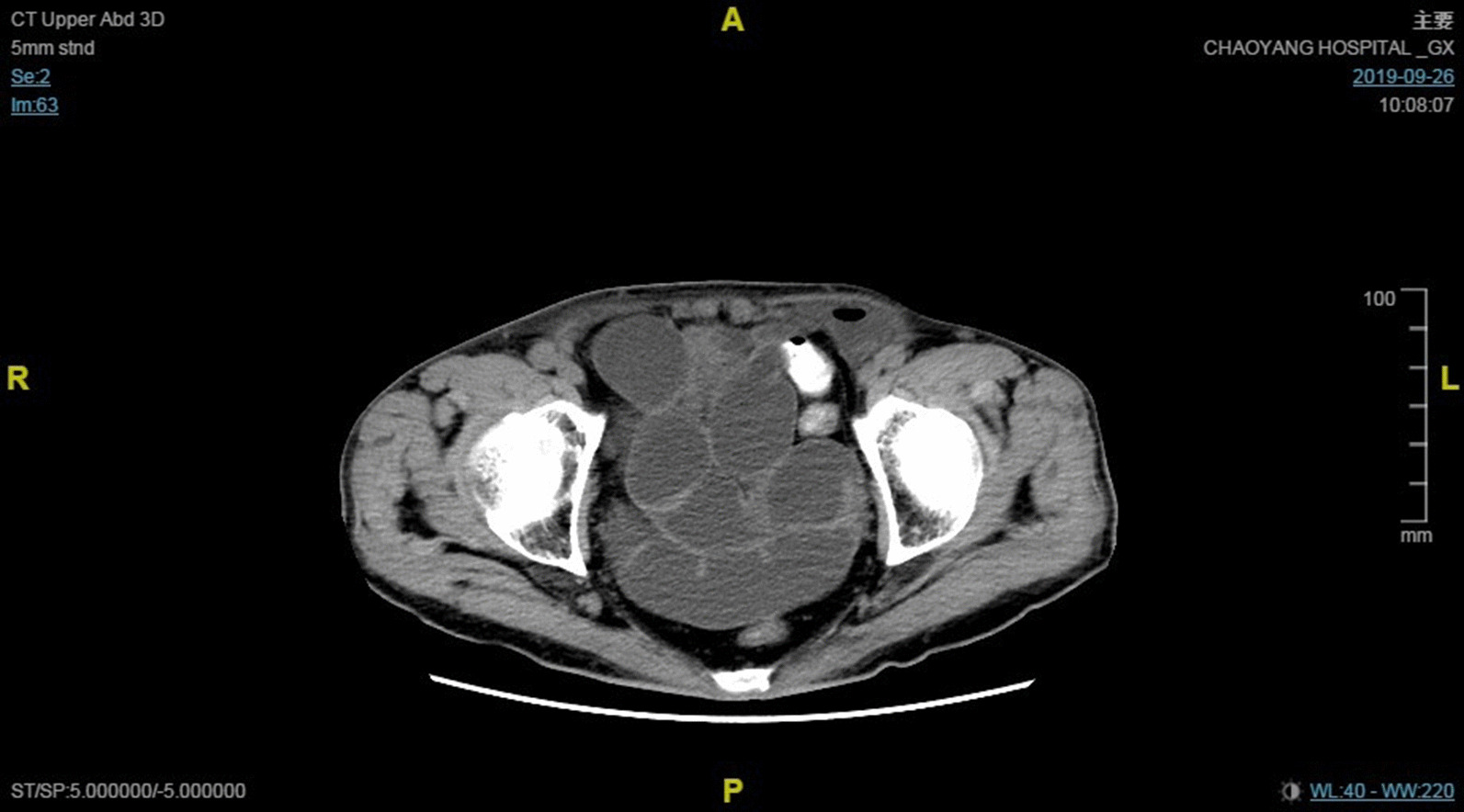
CT scans showed multiple distended bowel loops, exudates in both groin areas, and air-fluid levels in the left preperitoneal space

A 73-year-old man was admitted after having undergone bilateral TAPP with 3DMax mesh (C. R. Bard, Inc., Murray Hill, NJ, USA) at a secondary hospital 22 days previously. The mesh had been fixed by medical glue (Compont Medical Devices Co., Ltd., Beijing, China), and the peritoneal flap had been closed with interrupted suture. The patient had experienced abdominal distension since postoperative day 6. After conservative treatment, his symptoms did not substantially improve. CT scans showed multiple distended bowel loops, exudates in both groin areas, and air-fluid levels in the left preperitoneal space (Fig. 1).

**Fig. 2:**
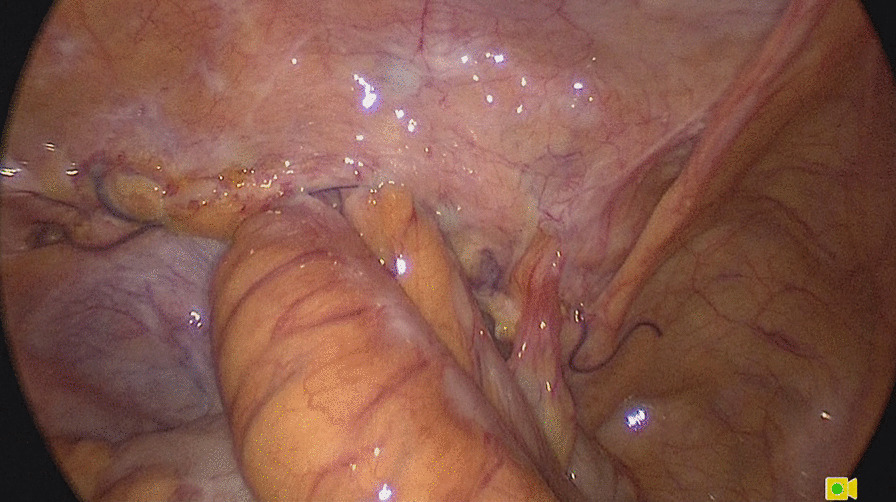
Small bowel loops herniated through a defect in the middle of the left peritoneum

**Fig. 3:**
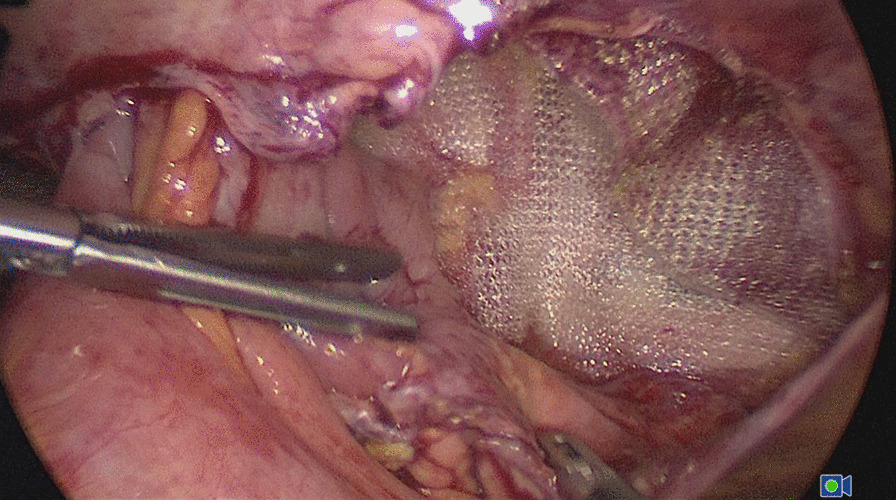
The small bowel loops were adhered to the mesh, and the medial part of the mesh was folded and displaced

**Fig. 4:**
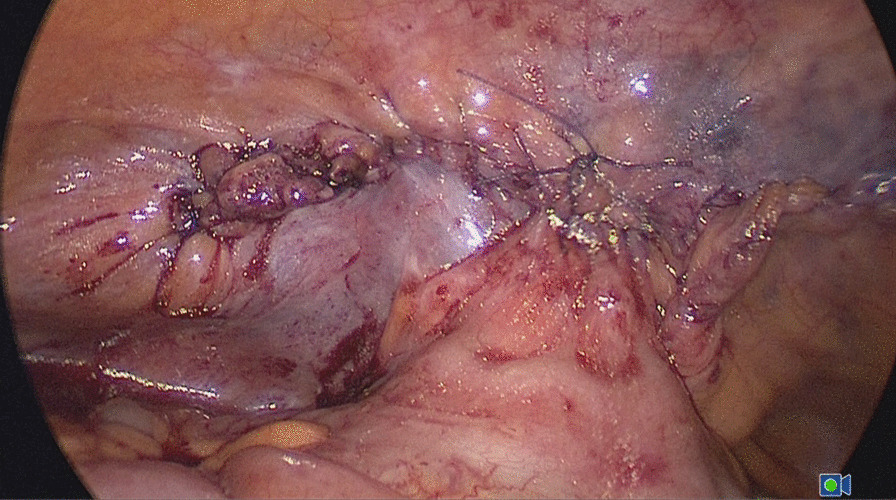
The peritoneal flap was closed with an absorbable running suture

Emergency laparoscopy was performed on the day of admission. The Hasson technique along the previous umbilical incision was used to enter the abdominal cavity. Assistant ports were placed under direct vision. Inspection of the abdominal cavity showed that small bowel loops had herniated through a defect in the middle of the left peritoneum (Fig. 2). The peritoneum was opened along the original incision. The small bowel loops were incarcerated in the preperitoneal space and adhered to the mesh, and the medial part of the mesh was folded and displaced (Fig. 3). All the small bowel loops were carefully dissected from the mesh and reduced. Part of the serosal layer was slightly injured without ischemia or perforation. The folded mesh was flattened and re-fixed by interrupted sutures. The pelvic cavity and the preperitoneal space were irrigated with sterile saline. A drainage tube was placed in the preperitoneal space. The peritoneal flap was closed with an absorbable running suture (Fig. 4). The patient passed flatus on the second postoperative day. He developed no further complications.

## Discussion and conclusions

TAPP achieves good results because its principle conforms to pathogenetic and pathophysiologic mechanisms of inguinal hernia. Specifically, in TAPP, the mesh is positioned between the origin of the pressure and the weak pelvic floor, and its mechanism is based on Pascal’s law [14]. Nevertheless, some serious and potentially life-threatening complications may develop. SBO after TAPP is uncommon, with an estimated incidence of 0.2–0.5% [10]. It usually results from inadequate peritoneal closure, trocar site herniation, or adhesion [10–12, 15]. PH caused by a disrupted peritoneum is extremely rare, but it will have serious consequences if not managed in a timely manner. Rodda et al. [16] first reported a complete mechanical obstruction produced by herniation through the stapled peritoneum. They observed a vacuum effect pulling the bowel into the preperitoneal space; this was caused by more rapid absorption of carbon dioxide in the preperitoneal space than in the abdominal cavity. The authors considered this to be a possible mechanism underlying the development of bowel obstruction.

In our center, more than 3000 inguinal hernia repairs are performed annually. LIHR accounts for more than 60% of these procedures. PH is extremely rare. In the first case of recurrence, a fusiform incision was made to open the peritoneum. Part of it remained on the surface of the plug, which resulted in higher tension during closure. In the second case, the peritoneum was closed by interrupted sutures. We believe that interrupted closure can be risky if a large gap is left. Therefore, the technique used to close the peritoneum is crucial.

Importantly, however, such a complication can be avoided. Any closure technique that is properly and skillfully performed in a strictly standardized way may achieve good or even excellent results [14]. We believe that closure of the peritoneum is one of the most challenging steps in TAPP, especially for surgeons at the beginning of the learning curve. Many methods for closure of the peritoneal flap exist, including the use of sutures, tacks, and staples. Improved postoperative activity and less pain are observed with suture closure; furthermore, the cost of sutures is much lower than the cost of tacks and staples [17]. Our practice is to close the peritoneal flap without any gaps using a running suture. Our suture technique has been highly effective for more than 10 years. Suturing begins from the right side of the peritoneal incision and is carried out from the lower flap to the upper flap using 3-0 Vicryl suture with a sleigh-shaped needle. This technique has been proven safe and has a shorter learning curve, shorter suture time, and less peritoneal tearing [18]. Several operative details related to reducing peritoneal tension are also important. First, the pneumoperitoneum pressure can be reduced to 8 to 10 mmHg when closing the peritoneal flap. Second, more peritoneum can be dissected from the cord structures downward to the peritoneal reflexion. Third, the peritoneal incision can be either fusiform or T-shaped according to the shape of the peritoneum that needs to be excised [19]. Finally, the carbon dioxide should be slowly released to avoid a sudden pressure difference between the abdominal cavity and the preperitoneal space.

Early diagnosis and proper treatment are the keys to a successful outcome in patients with post-TAPP SBO. The diagnosis of SBO is based on a clinical examination and routine radiological imaging. CT is necessary to distinguish the causes of obstruction, and its sensitivity and specificity may reach 90% [20].

Laparoscopy is recommended once SBO has been diagnosed. Its advantages are associated with its minimally invasive approach and include a reduced rate of complications, shorter hospitalization, and lower requirement for analgesics [20]. The trocar placement strategy is critical in patients undergoing repeat abdominal operations to avoid bowel injury. An ideal location for the initial trocar is away from the previous trocar sites. An open (Hasson) or closed (Veress) technique is suggested. The surgeon is recommended to use the technique with which he or she is most familiar. There is no evidence that one entry technique is superior or inferior to the other [14]. Inspection of the activity of the incarcerated bowel is mandatory and should be carefully performed. If intestinal necrosis or perforation occurs, the first consideration is whether the mesh is infected and whether it needs to be removed. Once mesh infection has occurred, treatment is a complicated and costly process. If mesh removal is desired, as much as possible should be removed while avoiding injury to important anatomical structures. The preperitoneal space and mesh should be irrigated, and a drainage tube should then be inserted. The peritoneum must be confirmed to be completely closed. If it cannot be closed, a biological mesh can be utilized as a peritoneum substitute, helping to prevent adhesion. Finally, it must be emphasized that comprehensive hernia-related or other postoperative complications should be reserved for experienced laparoscopic hernia surgeons.

In conclusion, SBO due to PH through a disrupted peritoneum is a rare but potentially fatal complication following TAPP. Technical considerations to avoid this complication include closure of the peritoneal flap and control of the operative details. Thorough knowledge of the anatomy, a detailed understanding of the pathophysiology of hernia disorders, and a strict standardization of the technique are of utmost importance.

## Data Availability

Not applicable.

## Abbreviations

LIHR: Laparoscopic inguinal hernia repair
SBO: Small bowel obstruction
TAPP: Transabdominal preperitoneal patch plasty
PH: Preperitoneal herniation
CT: Computed tomography
